# NMI: a potential biomarker for tumor prognosis and immunotherapy

**DOI:** 10.3389/fphar.2022.1047463

**Published:** 2022-11-23

**Authors:** Teng He, Yinbiao Qiao, Qi Yang, Jie Chen, Yongyuan Chen, Xiaoke Chen, Zhixing Hao, Mingjie Lin, Zheyu Shao, Pin Wu, Feng Xu

**Affiliations:** ^1^ Department of Infectious Diseases, The Second Affiliated Hospital, Zhejiang University School of Medicine, Zhejiang University, Hangzhou, China; ^2^ Division of Hepatobiliary and Pancreatic Surgery, Department of Surgery, The First Affiliated Hospital, Zhejiang University School of Medicine, Zhejiang University, Hangzhou, China; ^3^ Department of Emergency, The Second Affiliated Hospital, Zhejiang University School of Medicine, Zhejiang University, Hangzhou, China; ^4^ Department of Thoracic Surgery, The Second Affiliated Hospital, Zhejiang University School of Medicine, Zhejiang University, Hangzhou, China; ^5^ Key Laboratory of Tumor Microenvironment and Immune Therapy of Zhejiang Province, The Second Affiliated Hospital, Zhejiang University School of Medicine, Zhejiang University, Hangzhou, China

**Keywords:** NMI, pan-cancer, prognostic biomarker, immune infiltration, bioinformatics

## Abstract

N-Myc and STAT Interactor protein (NMI) is an interferon inducible protein participating in various cellular activities, and is widely involved in the process of tumorigenesis and progression. Studies have shown that the loss of NMI expression in breast cancer can promote its progression by inducing epithelial-mesenchymal transition (EMT). However, the expression level of NMI in other tumors and its impact on immune cell infiltration, patient prognosis, and drug treatment are still unclear. Here, we analyzed the role of NMI in pan-cancer through multiple omics data. We found that NMI was abnormally expressed in a variety of tumor tissues. The expression of NMI was closely related to the unique molecular and immunotyping, diagnosis and prognosis of various tumor tissues. In addition, we identified the main proteins that interact with NMI, and focused on the relationship between the clinical parameters of lower grade glioma (LGG) and NMI expression. Subsequently, we found that the expression of NMI was correlated with the infiltration of multiple immune cells and the expression of immune checkpoints. Finally, we also found that the expression of NMI was correlated with the sensitivity to multiple antitumor drugs. In conclusion, our comprehensive pan-cancer analysis of NMI revealed that it is a potential molecular marker for tumor diagnosis and treatment, plays an important role in tumor immunity, and is a promising molecular target for cancer treatment.

## Introduction

In recent years, the incidence rate of cancer has increased year by year, which has placed a serious burden on society (Ali et al., 2022). At present, cancer treatment mainly includes chemotherapy, surgery, radiotherapy, targeted therapy and immunotherapy. Although the treatment of cancer is constantly improving, the overall prognosis and survival of cancer patients have not significantly improved (Sung et al., 2021). Therefore, finding suitable biomarkers for tumor diagnosis and prognosis has become an urgent problem.

The N-Myc and STAT interactor (NMI) is encoded by the NMI gene and consists of a coiled coil domain (CC) at the N-terminus and two tandem domains (NID1, NID2) at the C-terminus, which can interact with transcription factors containing zip, helix-loop-helix (HLH) or HLH zip motifs (Zhu et al., 1999; Weng et al., 2022). Existing studies have shown that NMI plays an important role in the differentiation of the breast lumen (Pruitt et al., 2018) and the maintenance of alveoli (Alsheikh et al., 2021). The loss of NMI in breast cancer cells reduces their autophagy reactivity and chemosensitivity (Metge et al., 2015), and the recovery of NMI expression can be achieved by inhibiting the Wnt/β-catenin signaling pathway to play an antitumor role (Fillmore et al., 2009). Interestingly, NMI can also negatively regulate the expression of hTERT in breast cancer through the Yin Yang 1 (YY1) protein, to control tumor growth (Feng et al., 2017). In addition, Nagel et al. (Nagel et al., 2011) found that NMI was involved in the apoptotic activity of acute lymphoblastic leukemia. In a lung adenocarcinoma model, Wang et al. (Wang et al., 2017) found that NMI could inhibit multiple signaling pathways including p300-mediated NF- κB acetylation to control tumor growth. However, a recent study by Meng et al. (Meng et al., 2015) showed that high expression of NMI was significantly correlated with poor prognosis in glioblastoma multiforme (GBM) patients and was an independent risk factor for the prognosis of GBM patients. Subsequently, they also found that the expression of NMI was correlated with PTEN deletion and EGFR amplification in GBM patients.

Therefore, NMI may play different roles in different types of tumors, which may be caused by the heterogeneity of tumors. In addition, the specific mechanism of NMI and its correlation with immunotherapy need to be further elucidated. Therefore, we aimed to comprehensively evaluate the role of NMI at the pan-cancer level, establish the relationship between NMI expression and tumor diagnosis and prognosis, and determine the correlation between NMI expression and immune cell infiltration and immune checkpoint expression. Thus, this study provides new targets for cancer diagnosis, immunotherapy and prognosis.

## Materials and methods

### Gene expression analysis

NMI expression data in different types of normal tissues were obtained from the genotype-tissue expression (GTEx, http://commonfund.nih.gov/GTEx/) database. The expression data of NMI in tumor and normal tissues, and the clinical information of patients were obtained from The Cancer Genome Atlas (TCGA, http://cancergenome.nih.gov) and GTEx databases. NMI expression data in tumor cell lines were obtained from the Human Protein Atlas (HPA, https://www.proteinatlas.org/) database. The above data were analyzed by R software (version 3.6.3) and visualized by the ggplot2 R package. A *p* value <0.05 was set as the critical value and was considered statistically significant by the Wilcoxon test.

### Correlation analysis of NMI expression with tumor molecular and immune types

The correlation between the expression of NMI and the 6 immune types of tumors (C1 (wound healing); C2 (IFN-gamma dominant); C3 (inflammatory); C4 (lymphocyte depleted); C5 (immunologically quiet); C6 (TGF-β dominant) and their unique molecular types were obtained from the TISIDB (http://cis.hku.hk/TISIDB/) database.

### Functional enrichment analysis

The STRING database was used to analyze the top 50 proteins that strongly interact with NMI, and the parameters were set as follows: meaning of network edges: evidence, active interaction sources: Textmining, Experiments, Databases, minimum required interaction score: medium confidence (0.400). Subsequently, we performed Kyoto Encyclopedia of Genes and Genomes (KEGG) and Gene Ontology (GO) analyses of the above proteins using the clusterprofiler package and visualized them using the ggplot2 package.

### Analysis of the diagnostic and prognostic value of NMI

Receiver operating characteristic (ROC) curves were used to characterize the correlation between NMI expression and tumor diagnosis. Analysis was performed using the proc package and visualized using the ggplot2 package. An area under the curve (AUC) > 0.7 is regarded as having diagnostic accuracy, and an AUC >0.9 is regarded as having high diagnostic accuracy.

The value of NMI in the prognosis of tumor patients is characterized by its correlation with overall survival (OS), disease specific survival (DSS) and progression free interval (PFI). Correlation analysis of OS, DSS, PFI and clinical characteristics was performed in lower grade glioma (LGG) tumor types. Analysis was performed using the survival package and visualized using the survminer package. The median level of NMI expression was set as the cutoff value and statistical significance was tested by Cox regression analyses.

### Correlation between NMI expression and immune cell infiltration

The correlation between NMI expression and the infiltration of 28 immune cell subtypes was analyzed at the pan-cancer level by searching the TISIDB database. Subsequently, the correlation between the abundance of T cells, B cells, DCs, macrophages, CD8^+^ T cells, NK cells, cytotoxic cells and Treg cells and NMI expression in LGG was analyzed by the R package (GSVA). The Wilcoxon rank sum test was used for significance analysis.

### Correlation analysis of NMI expression with immune stimulatory and inhibitory molecules

The correlation between NMI expression and 45 immunostimulatory molecules and 24 immunosuppressive molecules was analyzed by the TISIDB database.

### Drug sensitivity analysis

We analyzed the relationship between the expression of NMI and the nomenclature of various types of antitumor drugs through the RNAactDrug database (http://bio-bigdata.hrbmu.edu.cn/RNAactDrug), and showed the top 20 drugs with the strongest correlation. Pearson and Spearman correlation methods were used to analyze the correlation.

## Results

### Analysis of gene expression

**FIGURE 1:**
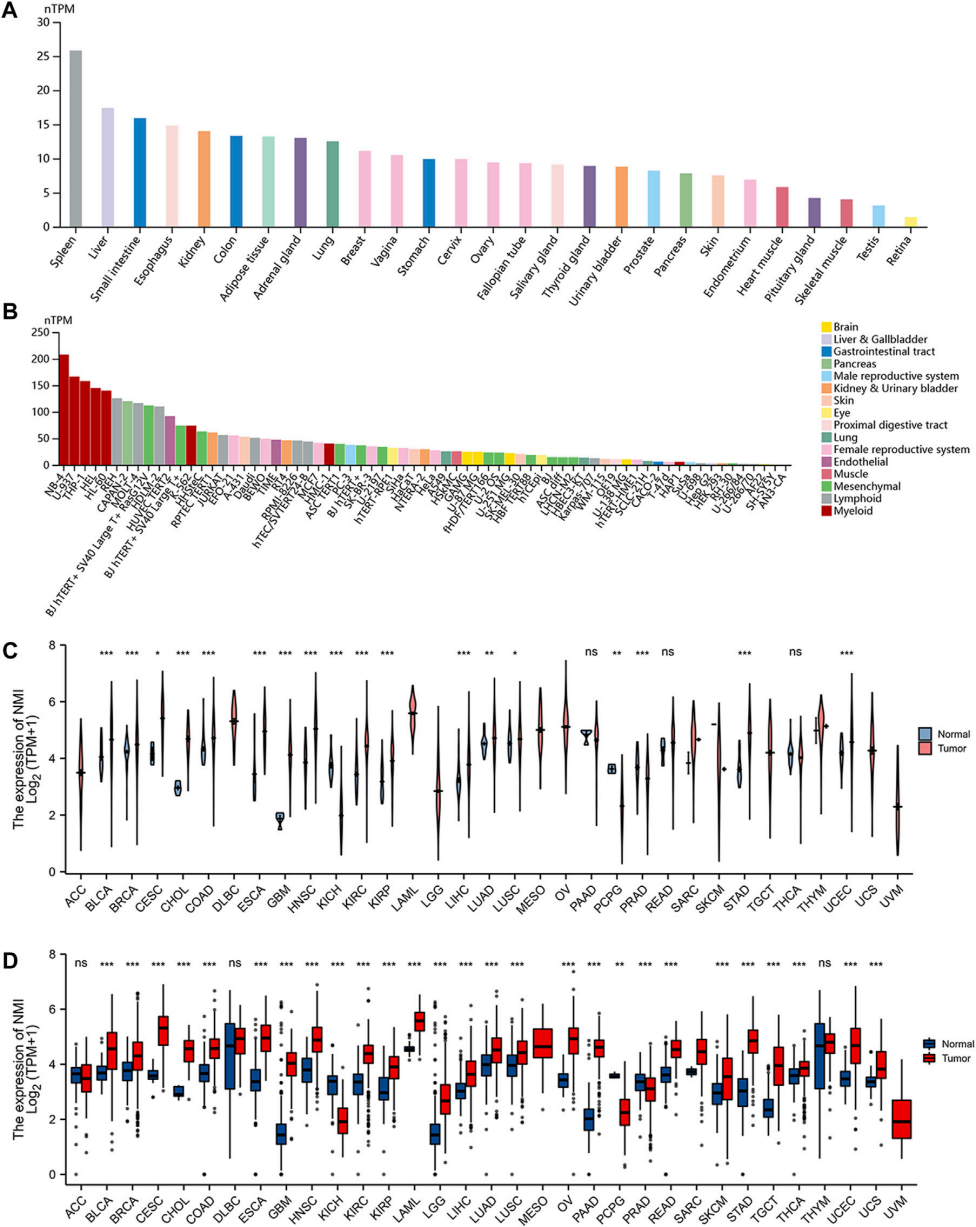
Expression level of NMI mRNA in pan-cancer. **(A)** NMI expression in normal tissues from GTEx database. **(B)** NMI expression in tumor cell lines from the HPA database. **(C)** Expression of NMI in tumor tissues and adjacent normal tissues from TCGA database. Pink represents tumor tissue and blue represents adjacent normal tissue. **(D)** Expression of NMI in tumor tissues and normal tissues in GTEx database. Red represents tumor tissue and blue represents adjacent normal tissue. *p < 0.05, **p < 0.01, ***p < 0.001. ns, not significant.

Using the GTEx database, we analyzed the expression of NMI in normal tissues. We found that NMI was expressed in most tissues and organs, and it was the most highly expressed in the spleen and liver (Figure 1A). Subsequently, by analyzing the HPA database, we found that NMI was also expressed in a variety of tumor cell lines, especially in bone marrow-derived tumor cells (Figure 1B). Compared with adjacent normal tissues, NMI was significantly upregulated in 15 tumor types, including bladder urothelial carcinoma (BLCA), breast invasive carcinoma (BRCA), cervical squamous cell carcinoma and endocervical adenocarcinoma (CESC), cholangiocarcinoma (CHOL), colon adenocarcinoma (COAD), esophageal carcinoma (ESCA), GBM, head and neck squamous cell carcinoma (HNSC), kidney renal clear cell carcinoma (KIRC), kidney renal papillary cell carcinoma (KIRP), liver hepatocellular carcinoma (LIHC), lung adenocarcinoma (LUAD), lung squamous cell carcinoma (LUSC), stomach adenocarcinoma (STAD) and uterine corpus endometrial carcinoma (UCEC), while it was downregulated in kidney chromophobe (KICH), pheochromocytoma and paraganglioma (PCPG) and prostate adenocarcinoma (PRAD) (Figure 1C). Compared with the normal organization of GTEx database, NMI was significantly upregulated in 24 tumor types, including BLCA, BRCA, CESC, CHOL, COAD, ESCA, GBM, HNSC, KIRC, KIRP, acute myeloid leukemia (LAML), LGG, LIHC, LUAD, LUSC, ovarian serous cystadenocarcinoma (OV), pancreatic adenocarcinoma (PAAD), rectum adenocarcinoma (READ), skin cutaneous melanoma (SKCM), STAD, testicular germ cell tumors (TGCT), thyroid carcinoma (THCA), UCEC and uterine carcinosarcoma (UCS). However it was downregulated in KICH, PCPG and PRAD (Figure 1D).

### Correlation analysis of NMI with tumor molecules and immune subtypes

**FIGURE 2:**
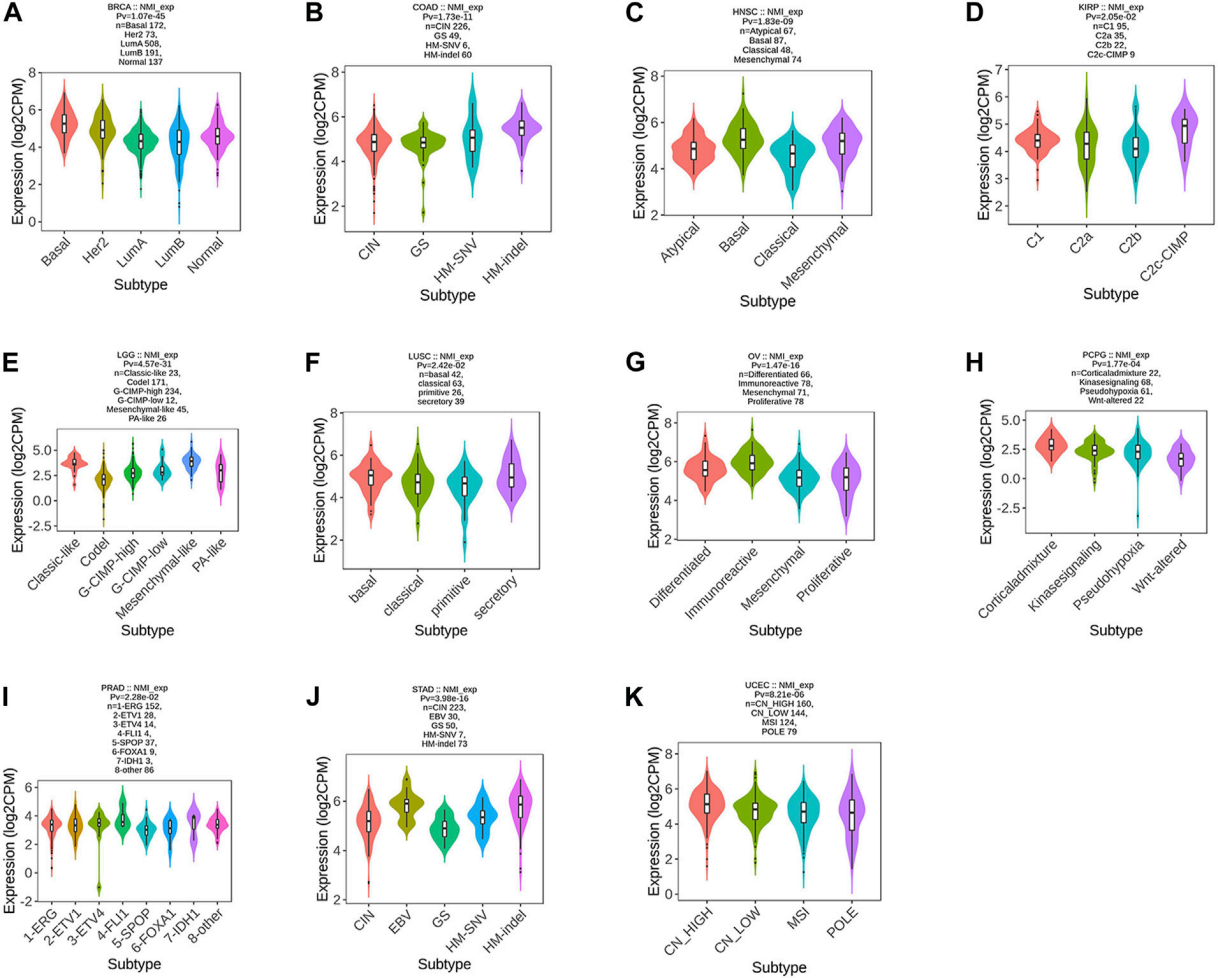
Correlation analysis between NMI expression and tumor molecular subtypes. **(A)** BRCA, **(B)** COAD, **(C)** HNSC, **(D)** KIRP, **(E)** LGG, **(F)** LUSC, **(G)** OV, **(H)** PCPG, **(I)** PRAD, **(J)** STAD, and **(K)** UCEC.

In the past, the classification of tumors was mainly based on pathological classification. With the rapid development of genomics and immunotherapy, molecular typing and immunotyping are urgently needed to guide the treatment of patients. Therefore, we first analyzed the correlation between NMI expression and tumor molecular type at the pan-cancer level. We found that the expression of NMI was mainly related to 11 types of tumors, including BRCA (Figure 2A), COAD (Figure 2B), HNSC (Figure 2C), KIRP (Figure 2D), LGG (Figure 2E), LUSC (Figure 2F), OV (Figure 2G), PCPG (Figure 2H), PRAD (Figure 2I), STAD (Figure 2J) and UCEC (Figure 2K).

**FIGURE 3:**
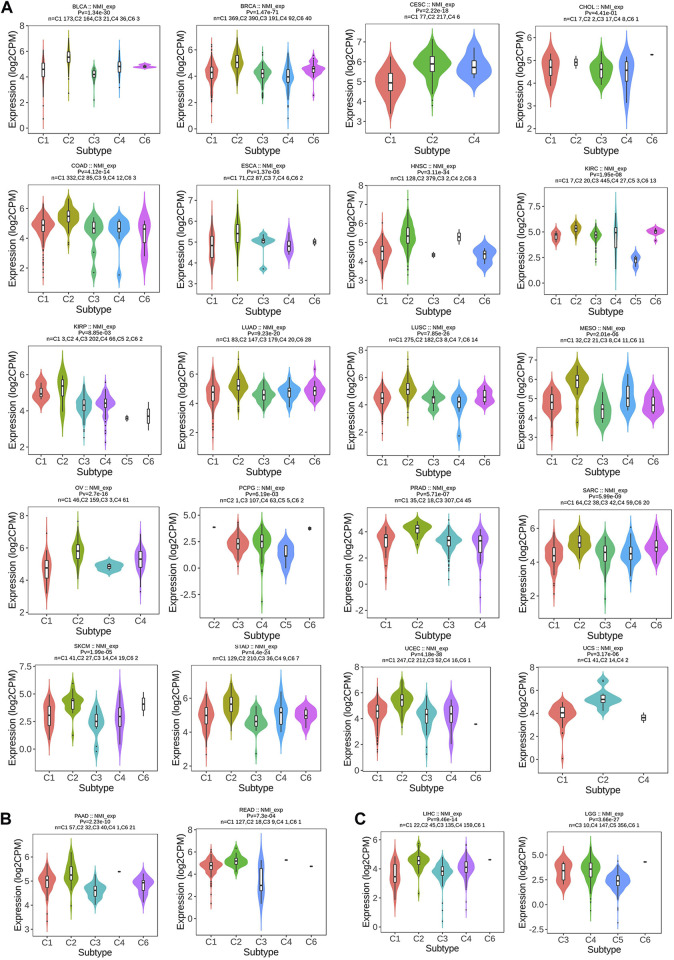
Correlation analysis between NMI expression and tumor immune subtypes. **(A)** BLCA, BRCA, CESC, CHOL, COAD, ESCA, HNSC, KIRC, KIRP, LUAD, LUSC, MESO, OV, PCPG, PRAD, SARC, SKCM, STAD, UCEC, and UCS. **(B)** PAAD and READ. **(C)** LIHC and LGG.

Next, we focused on analyzing the relationship between NMI expression and tumor immunotyping. We found that the expression of NMI was correlated with the immunotyping of 24 types of cancer. Among the 20 cancer types, high expression of NMI was mainly associated with C2 type, including BLCA, BRCA, CESC, CHOL, COAD, ESCA, HNSC, KIRC, KIRP, LUAD, LUSC, MESO, OV, PCPG, PRAD, SARC, SKCM, STAD, UCEC and UCS (Figure 3A). This suggests that the effect of NMI on tumorigenesis and progression may be mainly mediated by the expression of IFN-γ, which is also consistent with previous studies (Zhu et al., 1999). Interestingly, we observed that the highest expression of NMI was associated with C4 type (lymphocyte depleted) in PAAD and READ (Figure 3B), while the highest expression of NMI was associated with C6 type (TGF-β dominant) in LIHC and LGG (Figure 3C). This may be related to the small sample size of this subtype, but it also indicates that NMI may play different roles in different types of tumors.

### Functional enrichment analysis

**FIGURE 4:**
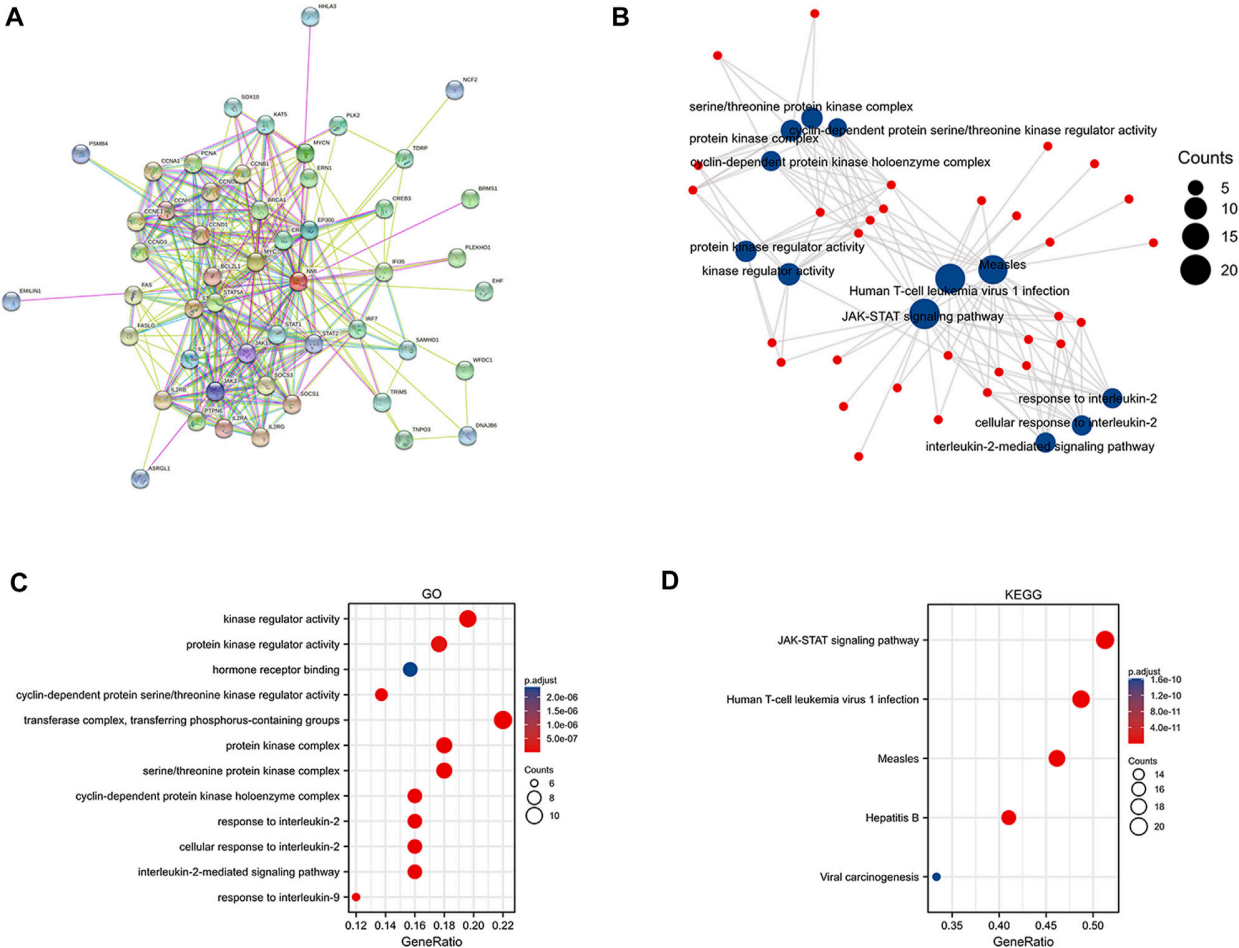
Functional enrichment analysis of NMI. **(A)** Protein network interaction diagram of NMI. **(B)** Scatter plot of GO and KEGG analyses of NMI interacting proteins **(C)** GO analysis of NMI-interacting proteins. **(D)** KEGG analysis of NMI-interacting proteins.

NMI proteins have attracted the attention of researchers because of their extensive interactions with transcription factors. Therefore, to further explore the protein interaction network of NMI, we analyzed the STRING database and obtained 50 proteins that closely interacted with NMI (Figure 4A). Subsequently, we performed KEGG and GO analysis of these proteins and visualized them (Figure 4B). GO enrichment analyses showed that NMI was mainly associated with “Kinase regulator activity”, “protein kinase regulator activity”, “transferase complex”, “transferring phosphorus-containing groups”, “protein kinase complex” and “serine/threonine protein kinase complex” (Figure 4C). KEGG enrichment analysis showed that NMI was mainly associated with “JAK-STAT signaling pathway”, “Human T-cell leukemia virus 1 infection”, “Measles” and “Hepatitis” (Figure 4D). Currently, many studies have established that NMI interacts with all signal transducers and activators of transcription (STATs) except STAT2 and enhances STAT-mediated transcription in response to the cytokines IL-2 and IFN-γ (Wang et al., 2017).

### The value of NMI in tumor diagnosis

**FIGURE 5:**
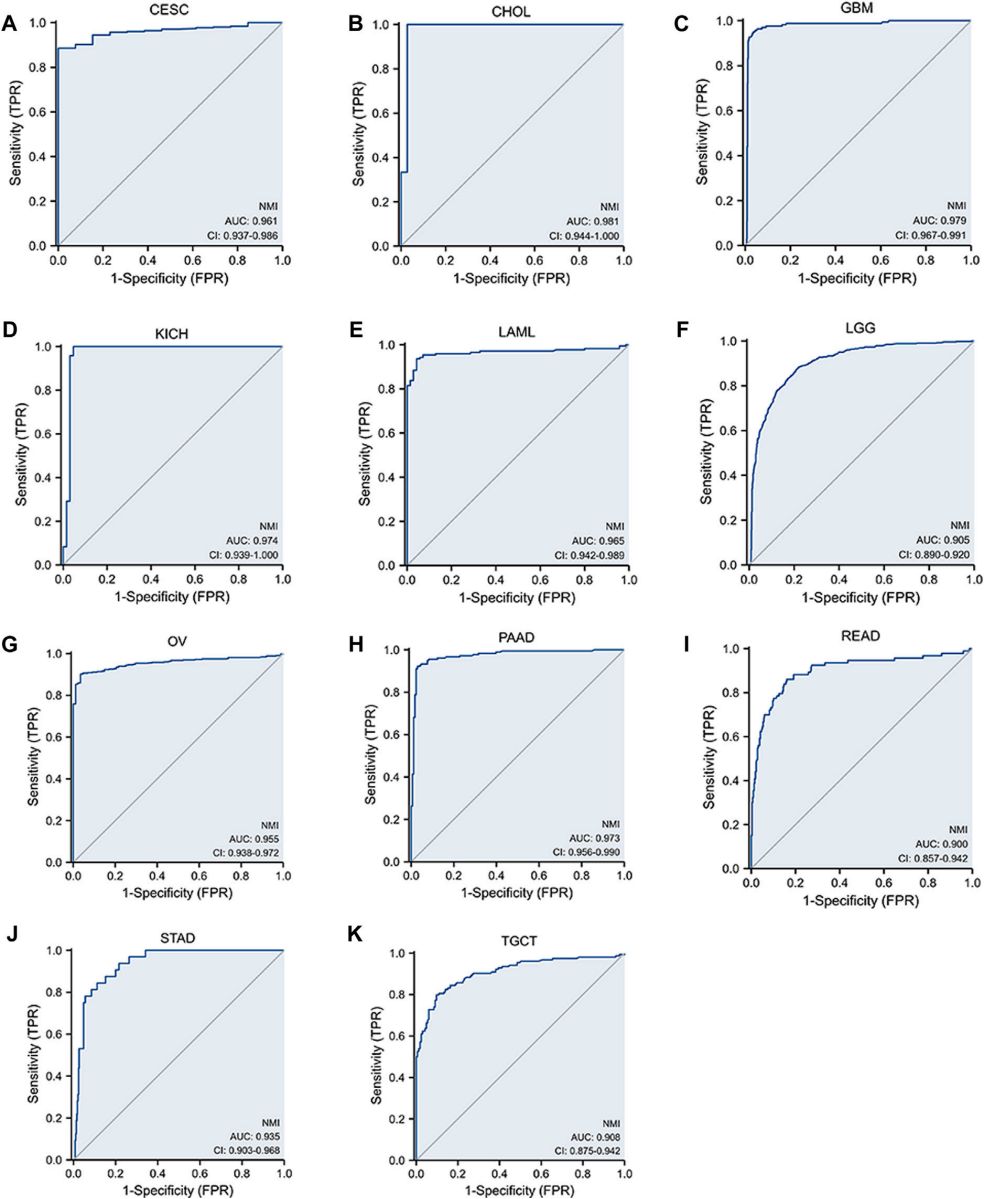
The sensitivity of NMI to tumor diagnosis is represented by the ROC curve. **(A)** CESC, **(B)** CHOL, **(C)** GBM, **(D)** KICH, **(E)** LAML, **(F)** LGG, **(G)** OV, **(H)** PAAD, **(I)** READ, **(J)** STAD, and **(K)** TGCT.

To further clarify whether NMI can be used as a biomarker for tumor diagnosis, ROC curves were used to explore the value of NMI in tumor diagnosis. We found that NMI accurately (AUC>0.7) predicted 8 types (Supplementary Figures S1A–H) and had higher accuracy (AUC>0.9) in 11 cancer types including CESC (AUC = 0.961) (Figure 5A), CHOL (AUC = 0.981) (Figure 5B), GBM (AUC = 0.979) (Figure 5C), KICH (AUC = 0.974) (Figure 5D), LAML (AUC = 0.965) (Figure 5E), LGG (AUC = 0.905) (Figure 5F), OV (AUC = 0.955) (Figure 5G), PAAD (AUC = 0.973) (Figure 5H), READ (AUC = 0.900) (Figure 5I), STAD (AUC = 0.935) (Figure 5J) and TGCT (AUC = 0.908) (Figure 5K).

### The value of NMI in tumor prognosis

**FIGURE 6:**
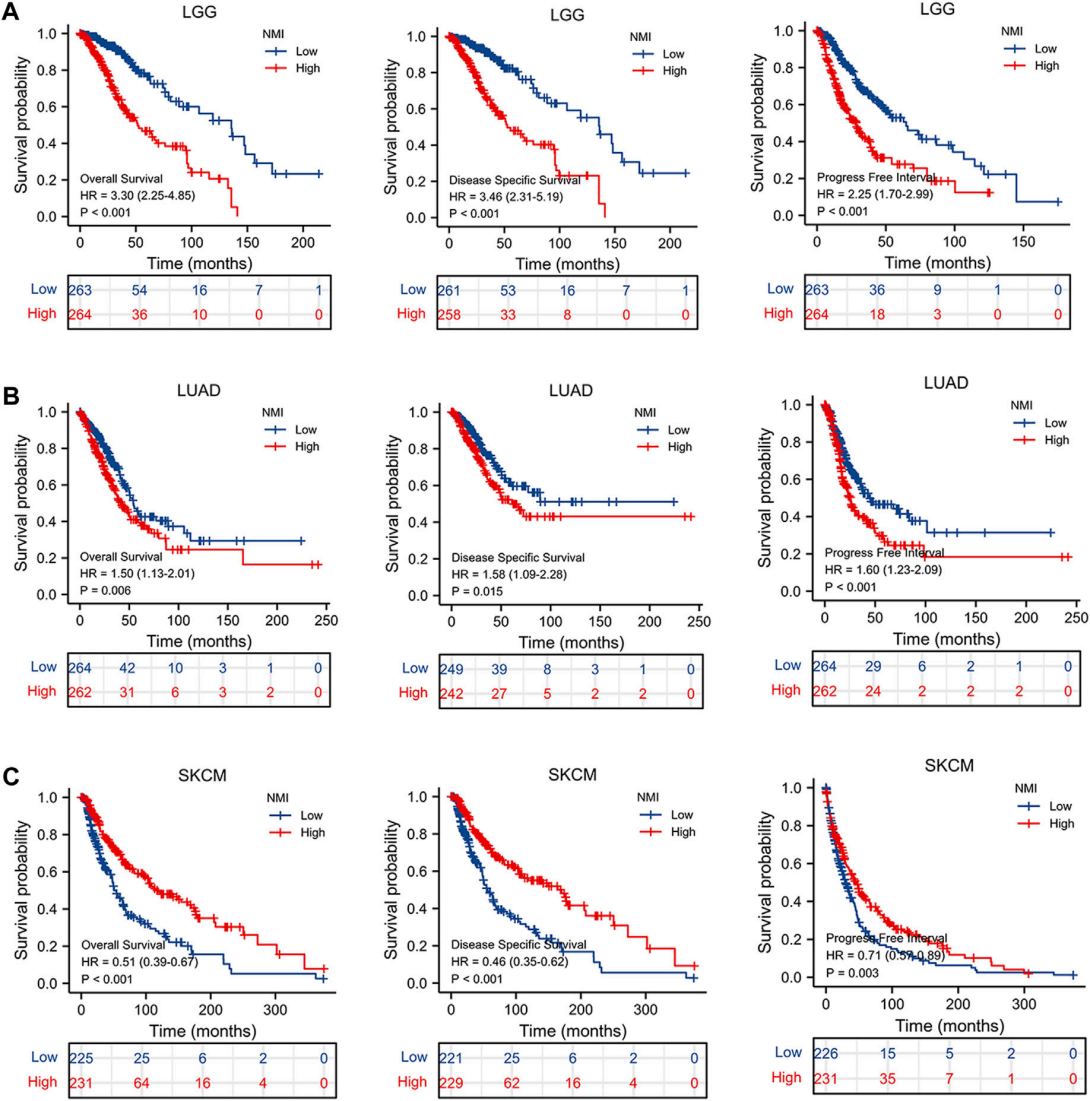
The relationship between NMI and OS, DSS and PFI in tumor patients. **(A)** LGG, **(B)** LUAD, and **(C)** SKCM.

To determine whether NMI has predictive value for tumor prognosis, we analyzed the relationship between NMI expression and the OS, DSS and PFI of tumor patients. We found that NMI was highly correlated with three prognostic indicators in LGG, LUAD and SKCM. For LGG, high expression of NMI was associated with worse OS (hazard ratio (HR) = 3.30, *p* < 0.001), DSS (HR = 3.46, *p* < 0.001) and PFI (HR = 2.25, *p* < 0.001) (Figure 6A). For LUAD, high expression of NMI was associated with worse OS (HR = 1.50, *p* = 0.006), DSS (HR = 1.58, *p* = 0.015) and PFI (HR = 1.60, *p* < 0.001) (Figure 6B). However, in SKCM, high expression of NMI was associated with better OS (HR = 0.51, *p* < 0.001), DSS (HR = 0.46, *p* < 0.001) and PFI (HR = 0.71, *p* = 0.003) (Figure 6C). In addition, the high expression of NMI was associated with poor PFI in ACC (HR = 3.52, *p* < 0.001) (Supplementary Figure S2A) and GBM (HR = 1.44, *p* = 0.041) (Supplementary Figure S2B) and better PFI in COAD (HR = 0.68, *p* = 0.029) (Supplementary Figure S2C) and READ (HR = 0.50, *p* = 0.04) (Supplementary Figure S2D). The high expression of NMI was also associated with poor OS (HR = 1.57, *p* = 0.032) and DSS (HR = 1.64, *p* = 0.038) of PAAD (Supplementary Figure S2E), poor OS (HR = 1.47, *p* = 0.029) of LIHC (Supplementary Figure S2F) and poor OS (HR = 0.40, *p* = 0.005) of osteosarcoma (Supplementary Figure S2G).

**FIGURE 7:**
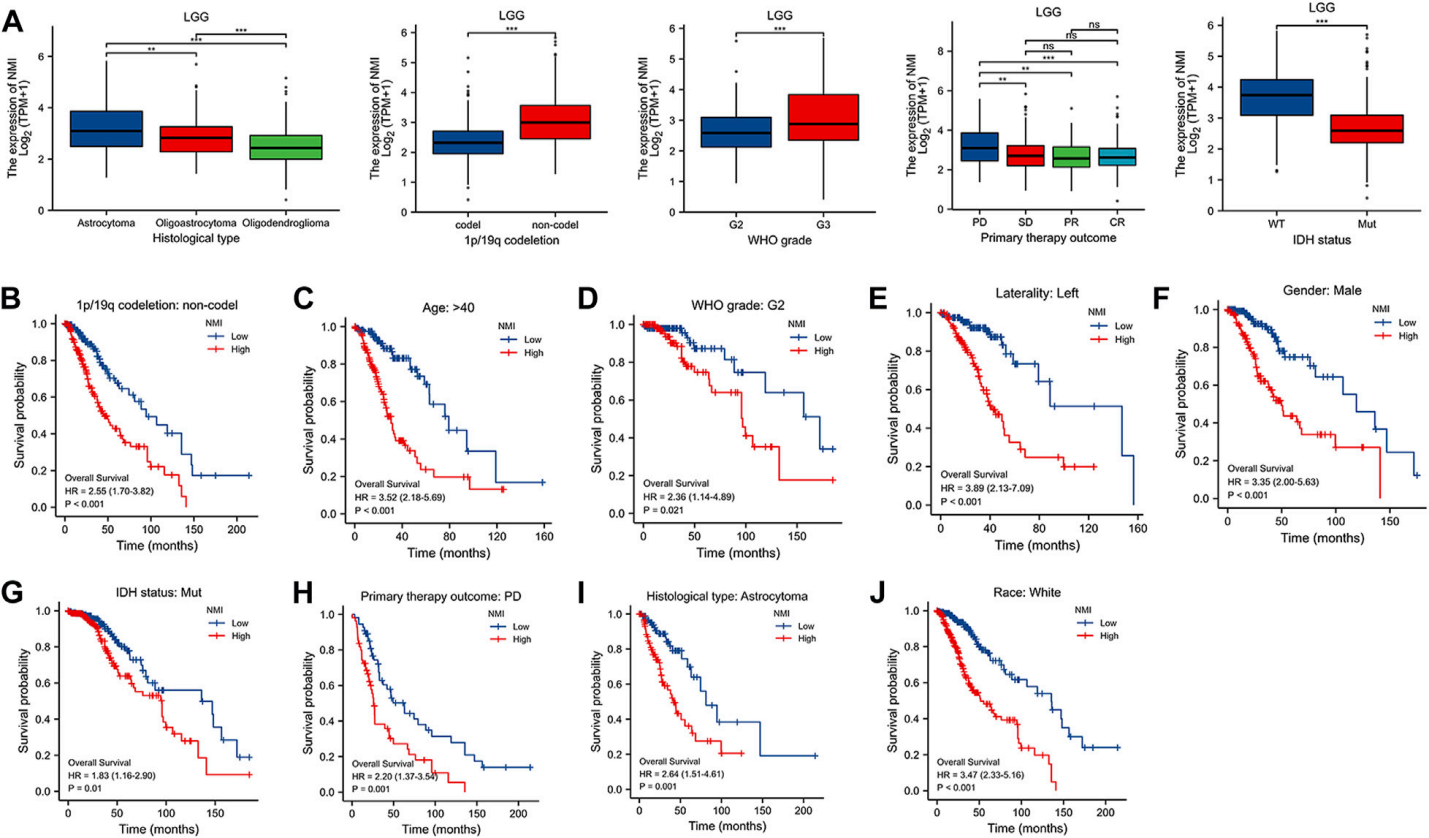
**(A)** Correlation between NMI and clinical features of LGG patients. **p < 0.01, ***p < 0.001. ns, not significant. **(B**–**J)** The relationship between the expression of NMI and OS in different clinical subtypes of LGG. **(B)** 1p/19q codeletion: non-codel, **(C)** age>40, **(D)** WHO grade: G2, **(E)** laterality: left, **(F)** sex: male, **(G)** IDH status: mutation, **(H)** primary therapy outcome: PD, **(I)** histological: astrocytoma, **(J)** race: white.

Through the above analysis, we found that the expression of NMI was significantly correlated with the diagnosis and prognosis of LGG patients. Therefore, we further analyzed the correlation between NMI expression and the clinical characteristics of LGG patients. We found that the expression of NMI was closely related to tumor histological type, 1p/19q codeletion, WHO grade, primary therapy outcome and isocitrate dehydrogenase (IDH) status (Figure 7A) but not to age, race, sex or laterality (Supplementary Figure S3A). In addition, we further explored the impact of NMI expression in different clinical subtypes on OS in LGG patients. The results showed that higher NMI expression was associated with worse OS in 1p/19q codeletion = non–codel (HR = 2.55, *p* < 0.001) (Figure 7B), age >40 years (HR = 3.52, *p* < 0.001) (Figure 7C), WHO grade = G2 (HR = 2.36, *p* = 0.021) (Figure 7D), laterality = left (HR = 3.89, *p* < 0.001) (Figure 7E), sex = male (HR = 3.35, *p* < 0.001) (Figure 7F), IDH status = mutation (HR = 1.83, *p* = 0.01) (Figure 7G), primary therapy outcome = progressive disease (PD) (HR = 2.2, *p* = 0.001) (Figure 7H), histological type = astrocytoma (HR = 2.64, *p* = 0.001) (Figure 7I), and race = white (HR = 3.47, *p* < 0.001) (Figure 7J).

### Correlation between NMI expression and immune cell infiltration

**FIGURE 8:**
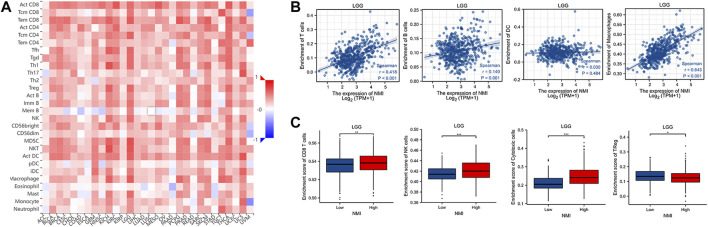
Correlation between NMI expression and immune cell infiltration. **(A)** The correlation heatmap between NMI expression at the pan cancer level and 28 immune cell subtypes was obtained from the TISIDB database. **(B)** Scatter plot of the correlation between NMI expression and T cells, B cells, DCs and macrophages in LGG. **(C)** In LGG, the correlation box diagram of NMI expression with CD8^+^ T cells, NK cells, cytotoxic cells and Treg cells. *p < 0.05, **p < 0.01, ***p < 0.001.

Immunotherapy is the fastest developing type of cancer treatment. Finding suitable biomarkers before and after treatment has become an urgent issue. A previous analysis suggested that NMI is closely related to the diagnosis and prognosis of tumor patients. We found that the expression of NMI was highly correlated with the abundance of various tumor infiltrating immune cells, especially in LGG, KICH, and UCS tumor types (Figure 8A). Next, we focused on analyzing the relationship between NMI expression and immune cell infiltration in LGG. The results showed that the high expression of NMI was accompanied by high infiltration of T cells, B cells and macrophages, but not DCs (Figure 8B). Interestingly, the expression of NMI was also associated with major innate and adaptive toxic cells (CD8^+^ T cells and NK cells) (Figure 8C). The high expression of NMI was accompanied by an overall increase in toxic cells, and the abundance of Treg cells decreased significantly.

### Correlation between NMI expression and immune checkpoint molecules

**FIGURE 9:**
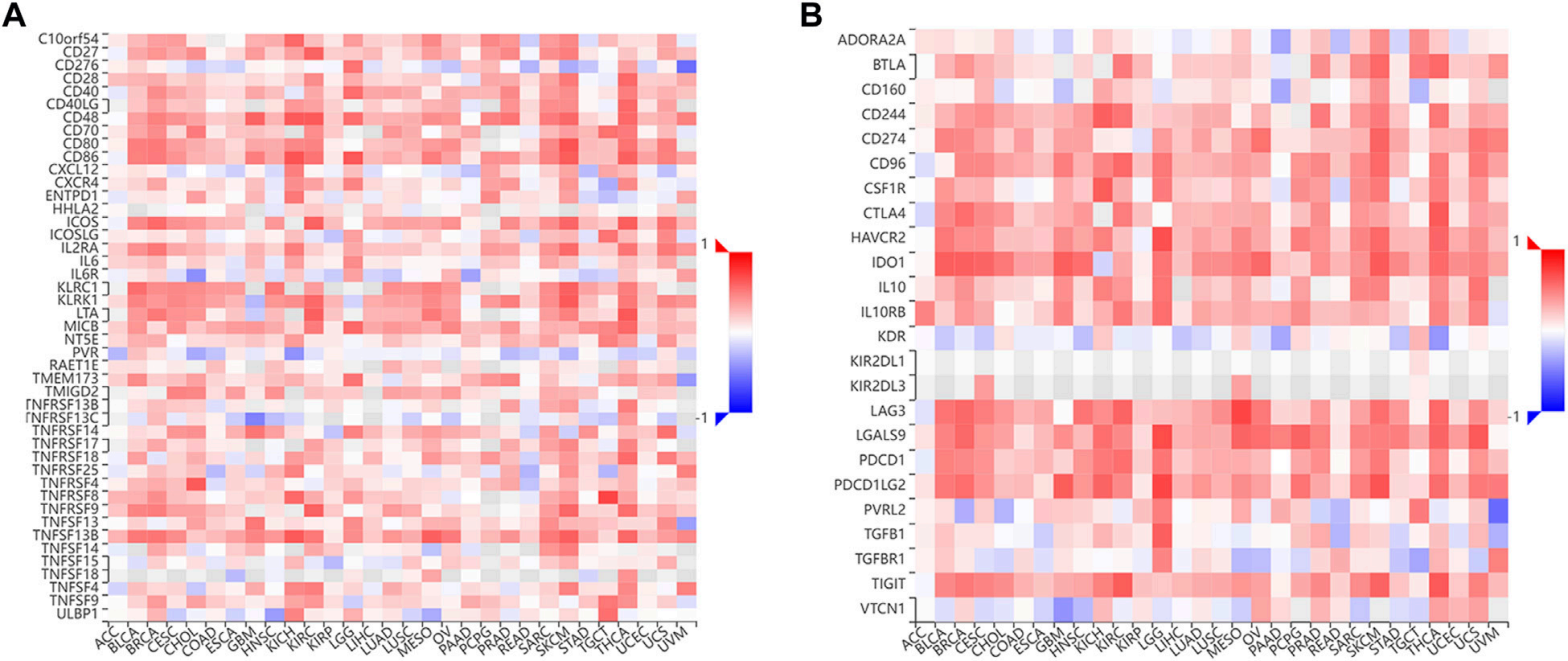
Correlation between NMI expression and immune checkpoint molecules. **(A)** The correlation heatmap between NMI expression and 45 immunostimulatory molecules was obtained from the TISIDB database. **(B)** The correlation Heatmap between NMI expression and 24 immunosuppressive molecules was obtained from the TISIDB database.

Immune checkpoint blockade (ICB) therapy is a new model of immunotherapy, that has profoundly changed the prognosis of many types of tumors. Therefore, we further explored the relationship between the expression of NMI and the expression of immune checkpoint molecules. We found that the expression of NMI was related to the expression of 45 immunostimulatory molecules, especially the immunostimulatory molecules CD28, CD40, ICOS and the CD28 ligand CD80/86 (Figure 9A). In addition, we also found that the expression of NMI was related to 24 immunosuppressive molecules, especially PDCD-1, LAG-3, CTLA-4, TIGIT and IDO1 (Figure 9B). This suggests that the abnormal expression of NMI may be a biomarker for ICB treatment.

### Drug sensitivity analysis

We explored the relationship between NMI mRNA expression and various types of antitumor drugs by searching the RNAactDrug database. We found that the top 20 drugs most closely related to NMI expression were Pcliainib, bosutinib, AICA Ribonucleotide, WHI-P97, idelalisib, BIX02189, TPCA-1, afatinib, SNX-2112, AT-7519, FR-180204, methotrexate, enzastaurin, OSU-03012, AZD7762, gemcitabine, AS605240, ZSTK474, CP466722 and VNLG/124 (Table 1).

**TABLE 1:** The correlation between NMI mRNA and anticancer drug sensitivity based on RNAactDrug data. ***p < 0.001.

Compound	RNAtype	RNAmoleade	Omits	Source	Peanon stat	Pearson.fdr	Spearmanstat	Spearman.dr
Pcliainib	mRNA	NMI	Expression	GDSC	−0.2490565	***	0.2946408	***
Bosutinib	mRNA	NMI	Expression	GDSC	−0.2363505	***	0.2504253	***
AICA Ribonueleotide	mRNA	NMI	Expression	GDSC	−0.1681221	***	0.2137404	***
WH1-P97	mRNA	NMI	Expression	GDSC	−0.1927539	***	0.2270336	***
Idelalisib	mRNA	NMI	Expression	GDSC	−0.2039266	***	0.2024053	***
B1X02189	mRNA	NMI	Expression	GDSC	−0.1779594	***	0.1952747	***
TPCA-I	mRNA	NMI	Expression	GDSC	−0.1651384	***	0.1916956	***
AICA Ribonueleotide	mRNA	NMI	Methylation	GDSC	0.14491277	***	0.20265865	***
Afatinib	mRNA	NMI	Expression	GDSC	0.1400821	***	0.1960697	***
SNX-2112	mRNA	NMI	Expression	GDSC	0.1475029	***	0.1897817	***
AT-7519	mRNA	NMI	Expression	GDSC	0.1375211	***	0.1839012	***
FR-180204	mRNA	NMI	Expression	GDSC	0.1633403	***	0.1868138	***
Methotrexate	mRNA	NMI	Expression	GDSC	0.1434948	***	0.1811458	***
Enzastaurin	mRNA	NMI	Expression	GDSC	0.1733262	***	0.1966106	***
OSU-03012	mRNA	NMI	Expression	GDSC	0.1685606	***	0.2017743	***
AZD7762	mRNA	NMI	Methylation	GDSC	0.14467147	***	0.19235985	***
Gcmciaabine	mRNA	NMI	Expression	GDSC	0.1880426	***	0.2103153	***
AS605240	mRNA	NMI	Expression	GDSC	0.1577792	***	0.1876233	***
ZSTK474	mRNA	NMI	Expression	GDSC	0.1659785	***	0.182499	***
CP466722	mRNA	NMI	Expression	GDSC	0.1495175	***	0.1771389	***

## Discussion

Tumorigenesis is a gradual process, involving changes in many genes and signal transduction pathways (Todorovic-Rakovic, 2022). Pan-cancer analysis can reveal tumor heterogeneity and provide options for finding personalized biomarkers and drug screening (Chen et al., 2022). Here, we analyzed the expression of NMI at the pan-cancer level and the relationship between the expression level and tumor molecules and immune typing through bioinformatics. Subsequently, we further evaluated the PPI of NMI and its relationship with tumor diagnosis, prognosis, immunotherapy and antitumor drug sensitivity.

Our results showed that NMI was expressed in a variety of normal tissues, but was significantly elevated in 24 tumor types and correlated significantly with molecular type in 11 tumor types. These results suggest that changes in NMI expression are deeply involved in tumorigenesis and progression. This also indicates that NMI may play different roles due to the heterogeneity of tumors.

In recent years, researchers have realized that type II interferon (IFN-γ) plays an important role in the tumor immune surveillance of tumors (Dunn et al., 2005). IFN-γ in tumor tissues is mainly produced by TILs, which can play the role of regulatory and cellular effector factors (Angelicola et al., 2021). IFN-γ can upregulate the expression of MHC-I molecules on the surface of tumor cells, inhibit the proliferation of tumor cells and promote their apoptosis (Chin et al., 1997; Hobeika et al., 1999; Castro et al., 2018). However, the activation of IFN-γ receptor (IFNGR) on tumor cells can mediate the upregulation of PD-L1 through the JAK/STAT signaling pathway (Ribas et al., 2015; Garcia-Diaz et al., 2017). IFN-γ can also promote tumor growth by affecting the number of vascular endothelial growth inhibitors (VEGIs) (Lu et al., 2014) and the infiltration of immunosuppressive cells (Nishibori et al., 2004). In our study, we found that the high expression of NMI was associated with the formation of C2 (IFN gamma dominant) type tumors. In addition, we observed that the expression of NMI was related to the C6 type (TGF-β dominant) of LGG. Studies have shown that TGF- β can promote the metastasis of glioma by upregulating synthesis and matrix metalloproteinase-2 (MMP-2), and downregulating metalloproteinase-2 (TIMP-2) (Zhang et al., 2021). TGF- β can also induce epithelial-mesenchymal transition (EMT) and stemness by enhancing the signal transduction of NF-κb and Wnt (Kashani et al., 2022). This may explain how the expression of NMI is related to the C6 type of LGG. Enrichment analysis also showed that NMI mainly affected the “JAK/STAT signaling pathway” and “kinase regulation activity”. This finding indicates that NMI may affect the JAK/STAT signaling pathway by regulating the expression of IFN-γ, thereby mediating the effect on tumorigenesis and progression. Moreover, NMI may play different roles in different types of tumors precisely because of the bidirectional regulatory effect of IFN-γ on tumors.

Adult glioma is a highly malignant tumor of the nervous system, that usually develops infiltratively (Figarella-Branger et al., 2011). According to the different types of origin, gliomas can be divided into three types: astrocytoma, oligoastrocytoma and oligodendroglioma (Davis, 2018). Common indicators for prognosis monitoring mainly include 1p/19q codeletion, IDH mutation, G-CIMP phenotype, TP53 mutation, and MGMT methylation (Baldock et al., 2014; Gusyatiner and Hegi, 2018; Guerrini et al., 2021). IDH mutation is currently recognized as an indicator of better prognosis. Our study found that the expression of NMI was negatively correlated with IDH mutation and 1p/19q codeletion, while high expression of NMI was associated with poor OS in LGG patients. Previous studies have shown that the high expression of NMI is related to the poor clinical prognosis of GBM patients, and the mechanism for this effect is that NMI regulates G1/S progression and cell proliferation through the interaction between STAT1 and NMI (Meng et al., 2015). Interestingly, we also found that the high expression of NMI was associated with the infiltration of toxic cells. This result may be related to that LGG with high NMI expression is more malignant, has higher heterogeneity, and can induce more immune cells to infiltrate, but the immune cells may be inhibited or exhausted.

Currently, immunotherapy is an important type of cancer treatment, that has profoundly changed the mode of cancer treatment (Zhu et al., 2021). However, there are still limitations in the effect of immunotherapy, which requires personalized selection of the treatment population (Zhang and Zhang, 2020). The foundation of immunotherapy is the number and status of infiltrating lymphocytes in tumor tissue, especially the existence of a depletion phenotype (Tan et al., 2020). In this study, we found that NMI is an important biomarker of immune cell infiltration and immune checkpoint expression. The expression of NMI was positively correlated with the number of infiltrating immune cells in most tumor tissues, especially the infiltration of tumor killer cells represented by CD8^+^ T cells and NK cells. In addition, the expression of NMI is also related to the expression of a variety of immune checkpoint molecules that have been widely studied. This provides an opportunity for us to accurately select the appropriate treatment population for ICB. Of course, our study still has limitations. Our results are based on the mRNA level and need further verification at the protein level. In addition, multicenter, large sample clinical studies are needed to further confirm this conclusion.

## Conclusion

Here, we revealed the expression changes of NMI in different tumor tissues, clarified its relationship with tumor molecules and immune typing, and confirmed that it can be used as a molecular marker for tumor diagnosis and prognosis. In addition, we also explored the relationship between NMI expression and immune cell infiltration and immune checkpoints, and identified it as a potential molecular marker for immunotherapy.

## Data Availability

The original contributions presented in the study are included in the article/Supplementary Material, further inquiries can be directed to the corresponding authors.
