# Supplementary material for: NMI: a potential biomarker for tumor prognosis and immunotherapy
Source: Front Pharmacol. 2022 Nov 23;13:1047463. doi: 10.3389/fphar.2022.1047463 (PMC9727384; doi:10.3389/fphar.2022.1047463)
Supplement: Supplementary file 1 [file DataSheet1.pdf]

Supplementary Materials for

NMI: a potential biomarker for tumor prognosis and immunotherapy

Teng He[1, †], Yinbiao Qiao[2, †], Qi Yang[3, †], Jie Chen[1], Yongyuan Chen[4, 5], Xiaoke Chen[4, 5], Zhixing Hao[4, 5], Mingjie Lin[4, 5], Zheyu Shao[4, 5], Pin Wu[4, 5, *] and Feng Xu[1, *]

[1]Department of Infectious Diseases, The Second Affiliated Hospital, Zhejiang University School of Medicine, Zhejiang University, Hangzhou, 310009, China

[2]Division of Hepatobiliary and Pancreatic Surgery, Department of Surgery, The First Affiliated Hospital, Zhejiang University School of Medicine, Zhejiang University, Hangzhou, 310009, China

[3]Department of Emergency, The Second Affiliated Hospital, Zhejiang University School of Medicine, Zhejiang University, Hangzhou, 310009, China

[4]Department of Thoracic Surgery, The Second Affiliated Hospital, Zhejiang University School of Medicine, Zhejiang University, Hangzhou, 310009, China

[5]Key Laboratory of Tumor Microenvironment and Immune Therapy of Zhejiang Province, The Second Affiliated Hospital, Zhejiang University School of Medicine, Zhejiang University, Hangzhou, 310009, China

*Correspondence: Feng Xu, xufeng99@zju.edu.cn and Pin Wu, pinwu@zju.edu.cn

Figure S1:

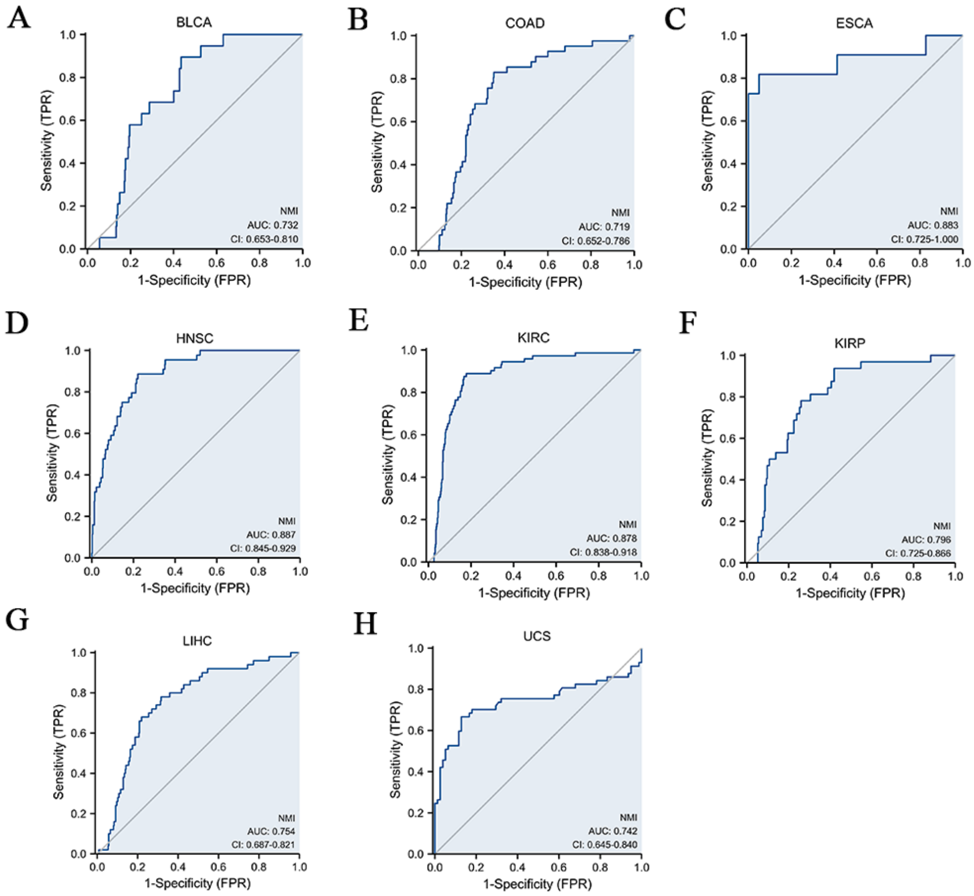

Figure S1: The sensitivity of NMI to tumor diagnosis is represented by ROC curve (AUC > 0.7). (A) BLCA, (B) COAD, (C) ESCA, (D) HNSC, (E) KIRC, (F) KIRP, (G) LIHC, and (H) UCS.

Figure S2:

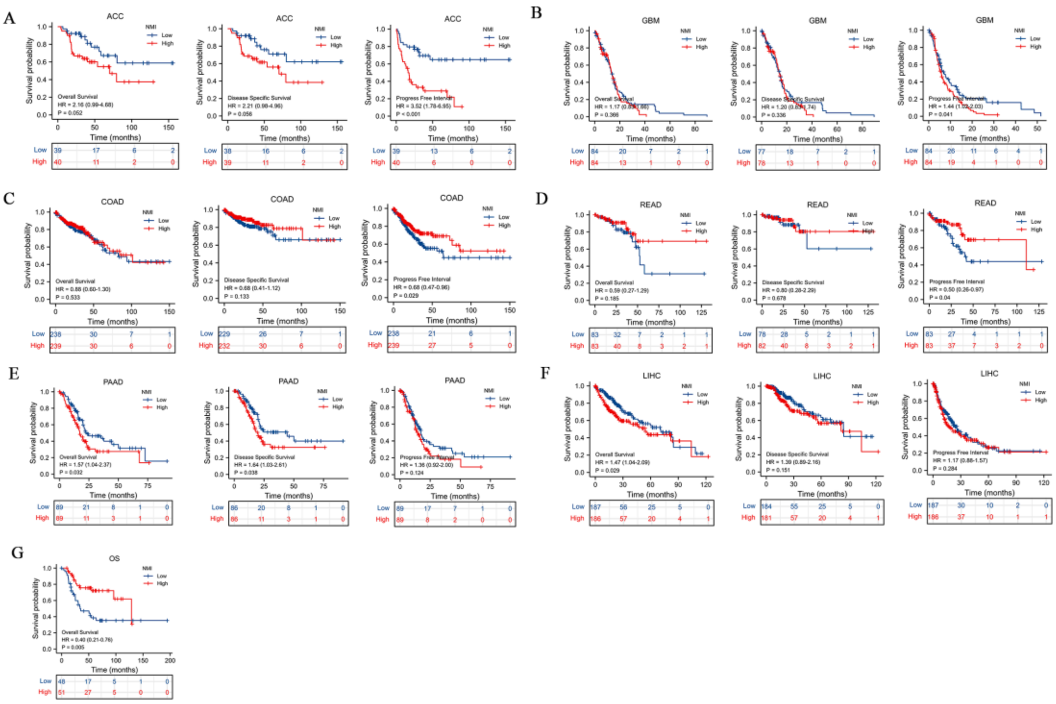

Figure S2: The relationship between NMI and OS, DSS and PFI in tumor patients. (A) Adrenocortical carcinoma (ACC), (B) GBM, (C) COAD, (D) READ, (E) PAAD, (F) LIHC, and (G) osteosarcoma.

Figure S3:

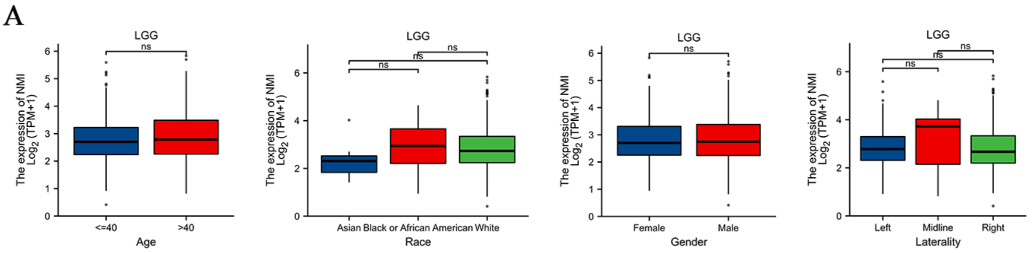

Figure S3: (A) Correlation between NMI and clinical features of LGG patients. ns, not significant.
